# Two Neural Circuits to Point Towards Home Position After Passive Body Displacements

**DOI:** 10.3389/fncir.2019.00070

**Published:** 2019-10-30

**Authors:** Jean Blouin, Anahid H. Saradjian, Jean-Philippe Pialasse, Gerome A. Manson, Laurence Mouchnino, Martin Simoneau

**Affiliations:** ^1^Aix-Marseille Univ, CNRS, Laboratoire de Neurosciences Cognitives, Marseille, France; ^2^Faculté de Médecine, Département de Kinésiologie, Université Laval, Québec, QC, Canada; ^3^Centre for Motor Control, University of Toronto, Toronto, ON, Canada; ^4^Centre Interdisciplinaire de Recherche en Réadaptation et Intégration Sociale (CIRRIS), Québec, QC, Canada

**Keywords:** idiothetic, vestibular, movement planning, frontal lobe, posterior lobe, space updating, body motion, human

## Abstract

A challenge in motor control research is to understand the mechanisms underlying the transformation of sensory information into arm motor commands. Here, we investigated these transformation mechanisms for movements whose targets were defined by information issued from body rotations in the dark (i.e., idiothetic information). Immediately after being rotated, participants reproduced the amplitude of their perceived rotation using their arm (Experiment 1). The cortical activation during movement planning was analyzed using electroencephalography and source analyses. Task-related activities were found in regions of interest (ROIs) located in the prefrontal cortex (PFC), dorsal premotor cortex, dorsal region of the anterior cingulate cortex (ACC) and the sensorimotor cortex. Importantly, critical regions for the cognitive encoding of space did not show significant task-related activities. These results suggest that arm movements were planned using a sensorimotor-type of spatial representation. However, when a 8 s delay was introduced between body rotation and the arm movement (Experiment 2), we found that areas involved in the cognitive encoding of space [e.g., ventral premotor cortex (vPM), rostral ACC, inferior and superior posterior parietal cortex (PPC)] showed task-related activities. Overall, our results suggest that the use of a cognitive-type of representation for planning arm movement after body motion is necessary when relevant spatial information must be stored before triggering the movement.

## Introduction

After exposure to passive rotation with the eyes closed, we have a fair idea of our new position relative to the surrounding objects. It would then be possible to move the arm to point towards these objects even without visual feedback. These perceptual and motor outcomes are thought to reflect the brain’s capacity to process idiothetic information generated during self-motion (e.g., from vestibular receptors) to update the spatial representation of the environment and to plan the arm motor commands.

Much progress has been made on the neural processes underlying spatial updating through idiothetic information since the seminal discovery of hippocampal place cells by O’Keefe and Dostrovsky (1971; for recent advances, see Cullen and Taube, 2017; Moser et al., 2017; Laurens and Angelaki, 2018). In contrast, the neural mechanisms underlying the control of movements through idiothetic information remain largely unknown. To help elucidate these mechanisms, we recorded human cortical activities associated with the planning of arm movements defined through self-motion sensory cues. We adapted an established protocol for investigating space updating processes in human and non-human primates by asking seated participants to move their arm with the same amplitude, but in the opposite direction, of the passive body rotation they just experienced (Bloomberg et al., 1988; Israël et al., 1993; Ivanenko et al., 1997; Blouin et al., 1998; Medendorp et al., 2002; Baker et al., 2003; Bresciani et al., 2005; Wei et al., 2006; Ventre-Dominey and Vallee, 2007; Simoneau et al., 2009). Relevant body rotation information for planning such movements are deemed to be largely mediated by vestibular inputs, as perception of passive motion in the dark is largely impaired in patients suffering total bilateral vestibular loss (Valko et al., 2012).

According to research on space perception, the parameters of the arm movement produced after body motion would be defined using a cognitive representation of the body-in-space position which is updated while being rotated (Loomis et al., 1996; Klier and Angelaki, 2008; Medendorp, 2011). As an important hub structure for processing visuospatial information (Andersen et al., 1997; Medendorp et al., 2003; Gutteling and Medendorp, 2016), the occipito-parietal cortex could then provide relevant information for planning the arm movement. This hypothesis is consistent with the observation made by Ventre-Dominey and Vallee (2007) that patients with an occipito-parietal lesion show large errors when pointing towards memorized targets after passive body motion.

The cingulate and prefrontal cortices, which both respond to vestibular inputs (Dieterich et al., 2003; Stephan et al., 2005; Smith et al., 2012), could also contribute to movement planning processes. The cingulate cortex indeed contains hand motor areas that connect to spinal motor neurons (Amiez and Petrides, 2014) and to the arm area of the motor cortex (Dum and Strick, 1991). The contribution of the prefrontal cortex (PFC) to vestibular-based motor processes is supported by the large errors observed in individuals with PFC lesions when producing vestibular memory-contingent ocular movements (Israël et al., 1992; Pierrot-Deseilligny et al., 1993; Pierrot-Deseilligny et al., 1995).

In the present study, we used the electroencephalographic (EEG) source localization to determine if the pattern of activation in the fronto-parietal areas would be consistent with the hypothesis that arm motor commands are built according to a cognitive representation of visual space that is updated during body rotations.

## Materials and Methods

### Participants

Nine healthy adults participated in this study (three women, mean age: 26.6 ± 2.7 years). All participants were right-handed with normal or corrected-to-normal vision. The experiment was conducted in accordance with the Declaration of Helsinki. A written informed consent was obtained from the participants before the study and the experiment was approved by the Laval University Biomedical Ethics Committee.

### Experimental Set-Up

We used the same experimental set-up that has been employed in other investigations of the vestibular functions (Simoneau et al., 2009; Mackrous and Simoneau, 2011, 2014, 2015). The participants sat on a chair in a completely dark room. They were secured to the chair using a four-point belt and the use of a chin rest limited movements of the head relative to the trunk during the chair rotations. The chair could be manually rotated around the vertical axis by an experimenter using a handle attached behind the chair. Rotating the chair manually minimized the risk of contaminating EEG recordings by electric noise that could be generated by motorized revolving chairs (for a discussion on this issue, see Nolan et al., 2009). An array of four LEDs placed on the floor behind the chair indicated to the experimenter the initial chair position and the angular targets of the rotations (i.e., 20°, 30° and 40° in the counter-clockwise direction). A laser fixed on the handle behind the chair and directed toward the array of LEDs helped the experimenter to produce the required chair displacements. The experimenter produced discrete chair motion without making corrections when the LEDs of the chair and of the angular target did not match. Note that the variability in the manually-produced rotations observed for each angular target, which was expected to be small according to previous studies from different laboratories using similar set-ups (e.g., Hanson and Goebel, 1998; Blouin et al., 2010; Mackrous et al., 2019), was not detrimental in the present experimental context. In fact, it introduced, together with the use of three different rotation amplitudes, uncertainty into movement planning and minimized the risk of participants implementing stereotyped arm motor responses. Chair angular position was recorded with an optical encoder (1 kHz, US digital, model H5S, Vancouver, WA, USA) fixed at the center of rotation of the chair (see below for chair kinematics analyses).

### Design of the Experiment

**Figure 1:**
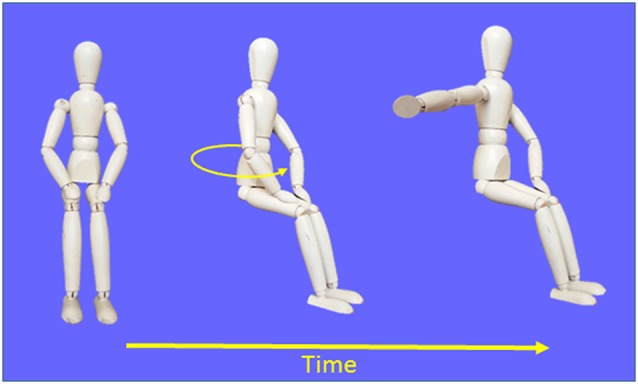
Schematic representation of the procedures used in the Movement condition.

Diagrams depicting the experimental task are provided in Figure 1. Throughout the trials, participants had to gaze at a chair-fixed LED located 1 m straight-ahead at eye level. This prevented participants from determining body displacements through sensorimotor signals linked to eye position and motion. Before each trial, the verbal instruction “ready” was given to the participants while their right hand was resting on their right thigh. Then, 2–3 s later, one of the three angular targets behind the chair turned on, indicating to the experimenter the rotation amplitude that had to be produced in the counter-clockwise direction. One-hundred milliseconds after rotation offset (i.e., after the chair angular velocity dropped below 2.5° s^−1^), a buzzer emitted a 50 ms tone. For the participants, this auditory cue served as an imperative (go) signal to reproduce the amplitude of perceived body rotations with a rapid horizontal movement of the right arm in the clockwise direction (i.e., opposite to the rotation direction). The movement consisted of external and upper rotations of the shoulder. Note that because the buzzer was fixed 1.5 m above the participants’ head, the tone could not be used as a spatial reference to determine body orientation after rotation. After the arm movement, the participants were rotated back to the starting position and got ready for the next trial which started only after a minimum delay of 15 s.

The EEG recordings after body rotations (i.e., during the planning of the arm movement) could contain noise and task-irrelevant activities induced for instance by the imperative auditory stimulus, residual rotation-induced ocular movements or artifacts arising from motion of the electrode cables. To identify the cortical activities related to arm movement planning, we compared the EEG recordings with those obtained in a control condition (referred to as no-movement condition) wherein no instruction other than keeping quiet during the rotation and after the tone (i.e., after rotation offset) were given to the participants. Our reasoning was that any differential activities after the tone between conditions with and without arm movement would reflect cognitive or sensorimotor processes relevant to movement planning. Participants performed 75 trials in both the movement and no-movement conditions (25 trials for each pseudo-randomly selected angular target) for a total of 150 trials. Five participants started the experimental session with the movement condition.

**Figure 2:**
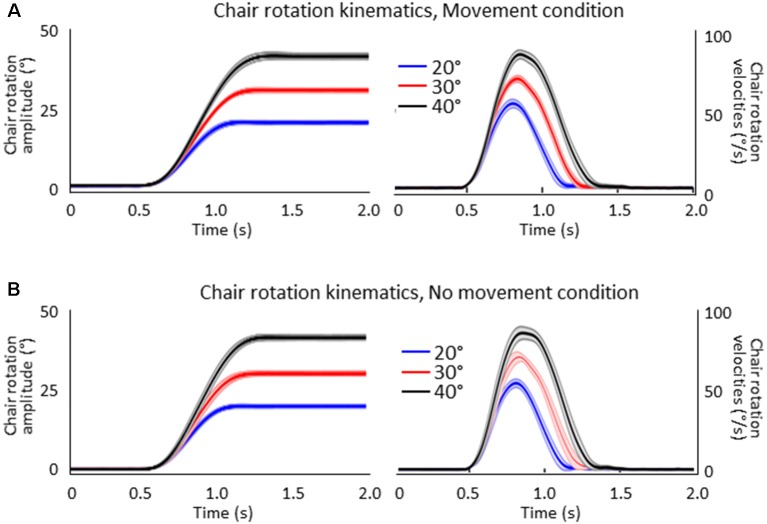
Mean amplitudes (left column) and velocities (right column) of the chair rotations for the 20°, 30° and 40° chair rotation conditions. Shaded areas represent the between-participant standard deviation of the means. **(A)** Movement condition. **(B)** No-movement condition.

Figure 2 shows the mean amplitudes (left column) and velocities (right column) of the chair angular displacement (with and without arm movements). The figure illustrates that, in both conditions, the body rotations had amplitudes close to the angular targets of the rotation (i.e., 20°, 30°, 40°) and had similar bell-shaped velocity profiles. Chair kinematics similarity between conditions was confirmed by the results of the ANOVAs 2 (Condition) × 3 (Angular rotation) which did not show significant effect (*p* > 0.05) of Condition on rotation amplitudes (*F*_(1,16)_ = 0.23, *p* = 0.64) or on peaks angular velocity (*F*_(1,16)_ = 0.85, *p* = 0.37) and neither an interaction of Condition × Angular rotation (*F*_(2,32)_ = 0.75, *p* = 0.48 and *F*_(2,32)_ = 1.96, *p* = 0.16, respectively). Overall, these results suggest that participants experienced similar idiothetic information when they produced or did not produce arm movements after the rotations.

### EEG Activity

EEG activity was recorded continuously at 1,000 Hz with a Geodesic 64-channel EEG sensor net (Electrical Geodesics Inc., Eugene, OR, USA). Data pre-processing was performed with BrainVision Analyzer 2 (Brain Products, Germany). The raw recordings were referenced to the averaged activity of the 64 electrodes before being synchronized with respect to the time of the auditory cue (i.e., the imperative signal in the movement conditions). Then, ocular artifacts (e.g., blinks, saccades) were subtracted from the EEG recordings by removing the corresponding component as revealed by the independent component analyses (ICA). In both conditions, we averaged the data for each participant and electrode. The EEG activities associated with the planning of the arm movement were therefore analyzed without considering the amplitude of the rotations that preceded the arm movements.

We estimated the neural sources of the late SEPs using the dynamical Statistical Parametric Mapping (dSPM, Dale et al., 2000) implemented in the Brainstorm software (Tadel et al., 2011, freely available at: http://neuroimage.usc.edu/brainstorm). We used the data from all processed sensors and averaged for each participant, condition, and electrode. The forward model was computed using a three-shell sphere boundary element model (BEM) on the anatomical MRI brain MNI Colin27 template (15,000 vertices), a predominant volume conductor model (Mosher et al., 1999; Huang et al., 2016). The baseline used to compute the co-variance matrices was set between −1.5 s and −1 s prior to the auditory cue, i.e., before the rotations as the longest recorded rotation duration was 1,490 ms.

**Figure 3:**
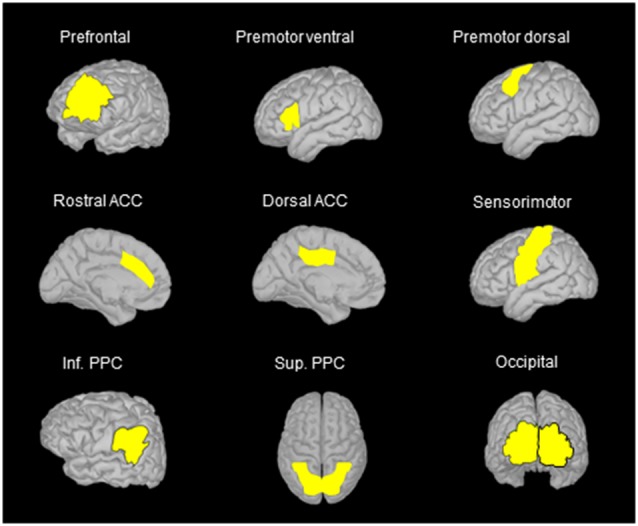
Location of the regions of interest (ROIs) on the anatomical MRI Colin 27 brain template that was used to compute cortical activations. The names of the ROIs were selected on the basis of the areas identified in classical cortical topographical maps that better represent the location of the ROIs used in the present study. Note that similar ROIs were defined for the left and right hemispheres, but only ROIs in the left hemisphere are illustrated when using side views. ACC, anterior cingulate cortex; Inf. PPC, inferior posterior parietal cortex; Sup. PPC, superior posterior parietal cortex.

Based on classical topographical maps of the cortex, we manually defined several region of interests (ROIs) in the frontal, parietal and occipital lobes. The location of these ROIs allowed us to assess the activation of regions that are deemed to be important for spatial representation and motor processes (see Figure 3). The number of vertices was similar for corresponding ROIs of the right and left hemispheres. In the movement condition, the mean absolute current amplitude (which reflects brain activation Tadel et al., 2011, 2019) was computed for each ROI between the auditory tone and the arm electromyography (EMG) onset (see below for EMG recordings). We operationally defined this time window as corresponding to the movement planning phase. Note however that processes related to motor planning might have occurred before the tone, for instance during body rotation, and that the activity recorded close to the movement onset could also be related to the commands triggering the arm movements.

The dynamics of the ongoing EEG activities was investigated by computing the mean absolute current amplitude during the planning phase for each ROI, for each quintile (see below “Muscle activity” section for quintile computation). The analysis of the ongoing cortical activity was made possible by the EEG’s excellent temporal resolution (Nunez and Srinivasan, 2006). It enabled us to determine if the activities computed in the different ROIs were more related to the generation of the motor commands (e.g., in case of a late increase of current amplitude) or rather to non-motoric processes (e.g., spatial or working memory processes, in case of an early or more sustained current flow).

### Behavioral Recordings

Hand movements were recorded at 100 Hz using a small (2.0 mm × 9.9 mm) 6-degree-of-freedom electromagnetic sensor (trackSTAR model 180, Ascension Technology Corporation, Shelburne, VT, USA) attached to the right fingertip. The norm of the vector between the initial and final hand position was respectively 32.55 ± 6.85 cm, 39.83 ± 6.92 cm and 48.19 ± 8.32 cm for the 20°, 30° and 40° body rotations. These observations indicate that participants scaled the amplitude of their movements according to the magnitude of their passive rotations as specified in the instructions.

### Muscle Activity

We recorded the EMG activity of the right posterior deltoid and of the triceps brachial muscles, which were the prime mover muscles for the required arm movements. After cleaning the skin with alcohol, we affixed self-adhesive bipolar Ag-AgCl electrodes (2 cm center-to-center inter-electrodes spacing) near the middle third of these muscles, along a line parallel to their fiber orientation (Brindle et al., 2006; Cram and Kasman, 2011). The EMG signals were pre-amplified (1,000×) at the skin site and then digitally sampled at 1 kHz using a Bortec AMT-8 system (Bortec Biomedical, Calgary, AB, Canada). The time elapsed between the imperative signal and the EMG onset was operationally defined as the planning phase. EMG onset was determined visually after summing the rectified EMG signals from both agonist muscles and squaring the results.

**Figure 4:**
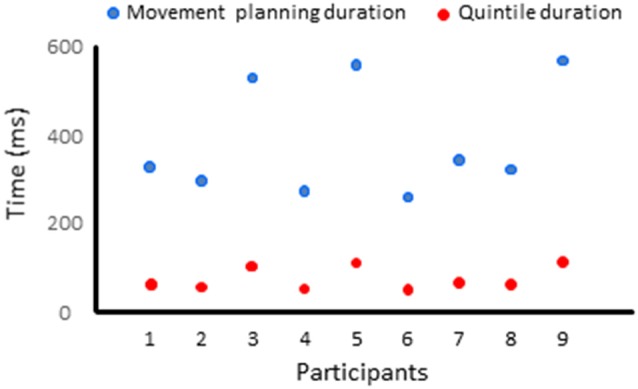
Duration of the movement planning phase and of the quintiles for each participant.

The movement planning phase was divided in quintiles, for each participant (i.e., the EMG reaction times were divided in five bins of equal duration). The duration of the quintiles depended on each participant’s average EMG reaction time (average EMG reaction times = 391 ± 127 ms, average quintile duration = 78 ms, see Figure 4). Quintiles of the same durations served to analyze the dynamics of the cortical activation for the Movement and No-movement conditions.

### Statistical Analyses

**Figure 5:**
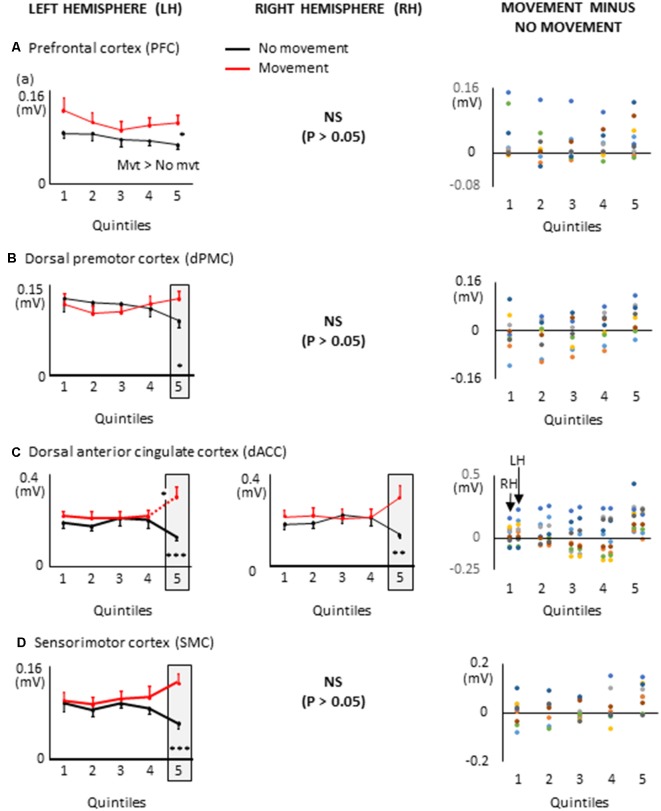
Mean activities recorded in the ROIs of the left and right hemispheres (left and middle columns, respectively) showing significant main effects of movement and significant interactions between Movement × Quintile in Experiment 1 [i.e., prefrontal cortex, **(A)**; dorsal premotor cortex, **(B)**; dorsal anterior cingulate cortex, **(C)** and sensorimotor cortex, **(D)**]. Error bars represent standard error of the mean. Dotted lines link consecutive quintiles showing significantly different activities (*p < 0.05, **p < 0.01, ***p < 0.001). Graphs of the right column depict the differences in activity between the Movement and No-movement conditions for all participants. Dots of the same color represent data from the same participant. Values greater than 0 (i.e., above the X-axis) indicate that the activity in the Movement condition was greater than the activity in the No-movement conditions. LH and RH indicate left and right hemispheres, respectively.

ANOVAs were used to contrast the mean current computed for each ROI between conditions with and without arm movements. All analyses employed a 2 (Condition: movement, no-movement) × 5 (Quintile: 1–5) design with repeated measures on both factors. As noted above, in all conditions, the EEG recording after the auditory signal (i.e., imperative signal in the movement condition) could contain noise or task-irrelevant activities that could decline with post-rotation duration. We thus reasoned that any differential activity between the movement and no-movement conditions would be specifically associated with task-relevant cognitive or sensorimotor processes. In this light, we will only consider here the significant main effects of Condition and the significant Condition × Quintile interactions as they were deemed to strictly reflect task-related processes. Significant Condition × Quintile interactions were further analyzed using Newman–Keul’s tests. Note that when main effects could be solely explained by a higher-order interaction, only the break-down of the interaction will be reported. Alpha level was set to 0.05 for all analyses. Table 1 reports all statistical results of the ANOVAs. Figure 5 depicts the results of the *post hoc* tests and the differences in activity between the Movement and No-movement conditions for all participants for ROIs showing significant effects.

**Table 1:** Results of the statistical analyses of Experiment 1 (No delay) and of Experiment 2 (Delay).

ROIs	No delay (Experiment 1)		Delay (Experiment 2)	
	Condition	Condition × Quintile	Task	Task × Quintile
Left PFC	*F*_(1,8)_ = 6.29; *p* = 0.03*	*F*_(4,32)_ = 0.67; *p* = 0.61	*F*_(1,8)_ = 12.56; *p* = 0.008**	*F*_(4,32)_ = 2.25; *p* = 0.08
Right PFC	*F*_(1,8)_ = 4.30; *p* = 0.07	*F*_(4,32)_ = 1.42; *p* = 0.25	*F*_(1,8)_ = 9.45; *p* = 0.01*	*F*_(4,32)_ = 0.87; *p* = 0.48
Left SMC	*F*_(1,8)_ = 2.65; *p* = 0.14	*F*_(4,32)_ = 3.95; 0.01*	*F*_(1,8)_ = 7.34; *p* = 0.02*	*F*_(4,32)_ = 3.23; *p* = 0.02*
Right SMC	*F*_(1,8)_ = 0.002; *p* = 0.96	*F*_(4,32)_ = 1.88; *p* = 0.14	*F*_(1,8)_ = 3.68; *p* = 0.09	*F*_(4,32)_ = 3.26; *p* = 0.02*
Left vPM	*F*_(1,8)_ = 0.09; *p* = 0.67	*F*_4,32_ = 0.59; *p* = 0.67	*F*_(1,8)_ = 5.36; *p* = 0.04*	*F*_(4,32)_ = 2.68; *p* = 0.04*
Right vPM	*F*_(1,8)_ = 0.01; *p* = 0.92	*F*_(4,32)_ = 0.24; *p* = 0.91	*F*_(1,8)_ = 0.21; *p* = 0.61	*F*_(4,32)_ = 0.30; *p* = 0.87
Left dPM	*F*_(1,8)_ = 0.001; *p* = 0.97	*F*_(4,32)_ = 4.77; *p* = 0.004**	*F*_(1,8)_ = 13.06; *p* = 0.007**	*F*_(4,32)_ = 6.44; *p* = 0.001**
Right dPM	*F*_(1,8)_ = 0.10; *p* = 0.755	*F*_(4,32)_ = 0.31; 0.87	*F*_(1,8)_ = 8.22; *p* = 0.02*	*F*_(4,32)_ = 2.63; *p* = 0.05
Left rACC	*F*_(1,8)_ = 3.44; *p* = 0.10	*F*_(4,32)_ = 1.62; *p* = 0.93	*F*_(1,8)_ = 18.26; *p* = 0.003**	*F*_(4,32)_ = 2.84; *p* = 0.04*
Right rACC	*F*_(1,8)_ = 1.90; *p* = 0.21	*F*_(4,32)_ = 1.28; *p* = 0.30	*F*_(1,8)_ = 17.13; *p* = 0.003**	*F*_(4,32)_ = 2.20; *p* = 0.09
Left dACC	*F*_(1,8)_ = 3.55; *p* = 0.09	*F*_(4,32)_ = 5.16; *p* = 0.003**	*F*_(1,8)_ = 11.58; *p* = 0.009**	*F*_(4,32)_ = 3.62; *p* = 0.01*
Right dACC	*F*_(1,8)_ = 2.11; *p* = 0.18	*F*_(4,32)_ = 4.04; *p* = 0.009**	*F*_(1,8)_ = 9.99; *p* = 0.01*	*F*_(4,32)_ = 2.76; *p* = 0.04*
Left iPPC	*F*_(1,8)_ = 1.47; *p* = 0.26	*F*_(4,32)_ = 1.98; *p* = 0.12	*F*_(1,8)_ = 13.7; *p* = 0.004**	*F*_(4,32)_ = 2.39; *p* = 0.07
Right iPPC	*F*_(1,8)_ = 0.15; *p* = 0.71	*F*_(4,32)_ = 1.02; *p* = 0.41	*F*_(1,8)_ = 0.23; *p* = 0.64	*F*_(4,32)_ = 7.77; *p* = 0.0001***
Left sPPC	*F*_(1,8)_ = 2.28; *p* = 0.16	*F*_(4,32)_ = 0.49; *p* = 0.74	*F*_(1,8)_ = 5.44; *p* = 0.04*	*F*_(4,32)_ = 3.96; *p* = 0.01*
Right sPPC	*F*_(1,8)_ = 0.51; *p* = 0.49	*F*_(4,32)_ = 2.06; *p* = 0.11	*F*_(1,8)_ = 3.90; *p* = 0.08	*F*_(4,32)_ = 4.69; *p* = 0.004**
Left Occip.	*F*_(1,8)_ = 0.0003; *p* = 0.98	*F*_(4,32)_ = 1.77; *p* = 0.16	*F*_(1,8)_ = 3.46; *p* = 0.10	*F*_(4,32)_ = 3.49; *p* = 0.01*
Right Occip.	*F*_(1,8)_ = 2.08; *p* = 0.19	*F*_(4,32)_ = 2.04; *p* = 0.11	*F*_(1,8)_ = 1.38; *p* = 0.27	*F*_(4,32)_ = 0.75; *p* = 0.56

*p < 0.05; **p < 0.01; ***p < 0.001.

## Results and Discussion

The ROIs showing significant movement-related activations were mainly circumscribed in the frontal lobe (Figure 5). The PFC contralateral to the reaching arm showed greater activation in the movement condition in all quintiles. The PFC is an important cortical region for stocking egocentric spatial information in working memory (Ma et al., 2012). The fact that the activity of the PFC was greater in movement condition from quintile 1 might suggest that information storage started during body rotations. The sustained activation found in the left PFC thus provides an electrophysiological support for the hypothesis issued from lesion studies that the PFC is involved in the storage of task-relevant vestibular signals (Berthoz et al., 1987; Pierrot-Deseilligny et al., 1993, 1995). On the other hand, the left dorsal premotor (dPMC) and sensorimotor (SMC) cortices, and the dorsal anterior cingulate cortices (dACC) of both hemispheres showed significantly greater activation in the movement condition only in the last quintile. The dPMC is known to contribute to the selection of motor responses that are based on spatial cues irrespective of the sensory modality of the cues (Weinrich and Wise, 1982; Wise, 1985). Thus, the increased activation of the dPMC during movement planning could be linked to the sensorimotor processes associated with the processing of vestibular spatial cues. Furthermore, the increased activation of both the dACC and SMC were expected near the end of movement planning, as these regions are important sources of descending motor commands. The bilateral increased activation of the dACC is also consistent with the existence of direct bilateral connections of this motor area of the medial wall with the spinal cord and the primary motor cortex (He et al., 1995; Dum et al., 2016). The motor commands issued from the dACC may have benefited from relevant spatial information stored in the PFC which has dense interconnections with the dACC (Yeterian et al., 2012). Note that the activations of the SMC and dACC could also be linked to the anticipatory postural adjustments observed before rapid arm movements (e.g., Massion, 1994; Kurtzer et al., 2005).

Remarkably, critical regions for the cognitive encoding of space, such as the ventral premotor cortex (vPM) or the posterior parietal cortex (PPC), did not show significantly different activities between conditions with and without arm movements. This result is not consistent with the scheme that movement planning was based on a cognitive representation of space updated during body rotations. Rather, the observation that task-related activities were exclusively found in PFC, dPM, dACC and SMC points to a dominant role of sensorimotor-type of spatial representations for converting self-motion sensory cues into arm motor commands. The short time elapsed between the end of the rotation and the onset of the arm muscular activity (i.e., 391 ms, see “Materials and Methods” section) is in line with the use of such sensorimotor representations wherein encoded information is short-lived.

Indeed, the motor representations of space rapidly decay when sensory stimuli relevant for triggering motor action becomes unavailable (Bridgeman, 1991; Goodale et al., 1994; Rossetti, 1998; Burgess, 2008; Ball et al., 2010). Therefore, the contribution of cortical regions involved in the cognitive encoding of space, including those tightly linked to spatio-motor integration, could increase for planning movements when there is a delay between body motion and the goal-directed arm movement. We tested this hypothesis in Experiment 2.

## Experiment 2

Nine new participants participated in Experiment 2 (three women, mean age: 25.5 ± 3.4). A written informed consent was obtained from the participants before the study and the experiment was approved by the Laval University Biomedical Ethics Committee.

### Design of the Experiment

This experiment reproduced the movement condition of Experiment 1 with the only exception being that the imperative signal prompting the participants to produce the arm movement occurred 8 s after the end of the chair rotation. This delay was chosen based on previous studies showing that most motor actions rely on a cognitive representation after a 8 s delay (Bridgeman, 1991; Gentilucci et al., 1996). As in Experiment 1, participants performed 25 trials for each of the three pseudo-randomly presented angular rotations (i.e., 20°, 30° and 40°, total of 75 trials). The kinematics of the chair rotations were like those of Experiment 1. On average, the rotation amplitudes were 20.16 ± 0.46°, 29.96 ± 0.34° and 40.43 ± 0.35° and their respective peak angular velocities were 58.10 ± 6.34°/s, 75.31 ± 7.49°/s and 92.59 ± 5.98°/s. Similarities of the chair rotation kinematics between both experiments were confirmed by the results of the mixed-design ANOVAs [3 (Condition: movement Experiment 1, no-movement Experiment 2) × 3 (Amplitude: 20°, 30°, 40°)] that did not reveal significant effect of Condition on the amplitude of the rotations (*F*_(2,24)_ = 1.79, *p* = 0.19) or on the chair peak angular velocities (*F*_(2,24)_ = 0.48, *p* = 0.62).

The norms of the vectors between the initial and final hand positions were also scaled in Experiment 2 to the amplitude of the chair rotations (i.e., 31.26 ± 6.56 cm, 37.42 ± 6.83 cm and 45.23 ± 5.88 cm, for the 20°, 30° and 40° body rotations, respectively). Moreover, the results of a 2 (Experiment) × 3 (Angular amplitude) mixed-design ANOVA showed that the norms of the movement vectors were not significantly different between both experiments (no significant main effect of Experiment (*F*_(1,14)_ = 0.43, *p* = 0.52).

The fact that participants remained motionless while waiting for the imperative signal which occurred 8 s after rotation offsets, minimized the possibility of contamination of the EEG recordings from residual eye movements or cable motion as in Experiment 1. However, the response to the auditory stimulation and the non-motoric activity related to anticipation (Simons et al., 1979) and expectancy (Ruchkin et al., 1986) of this stimulation could affect the post imperative signal cortical activity. To control for this possibility, we contrasted the recorded EEG activity with the EEG activity recorded after the second of two tones interspaced by 3 s that were presented in a control condition without body rotation and arm movement. Note that the first tone in this control condition and the end of the rotation in the delayed movement condition both served as a preparatory pre-cue signal as they were both followed by a tone that occurred after a fixed interval. In this control condition, each participant also performed 75 trials. Five participants started the experimental session with the delayed movement condition.

**Figure 6:**
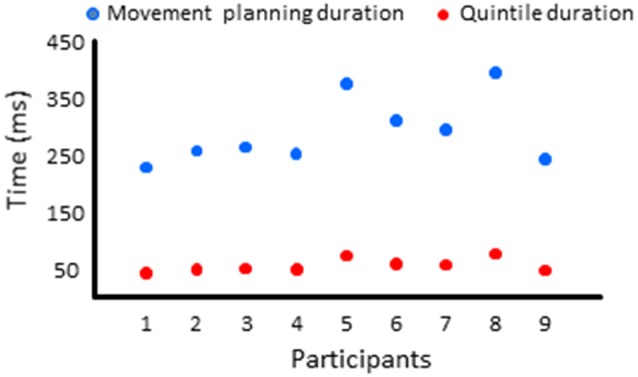
Duration of the movement planning phase and of the quintiles for each participant in Experiment 2.

**Figure 7:**
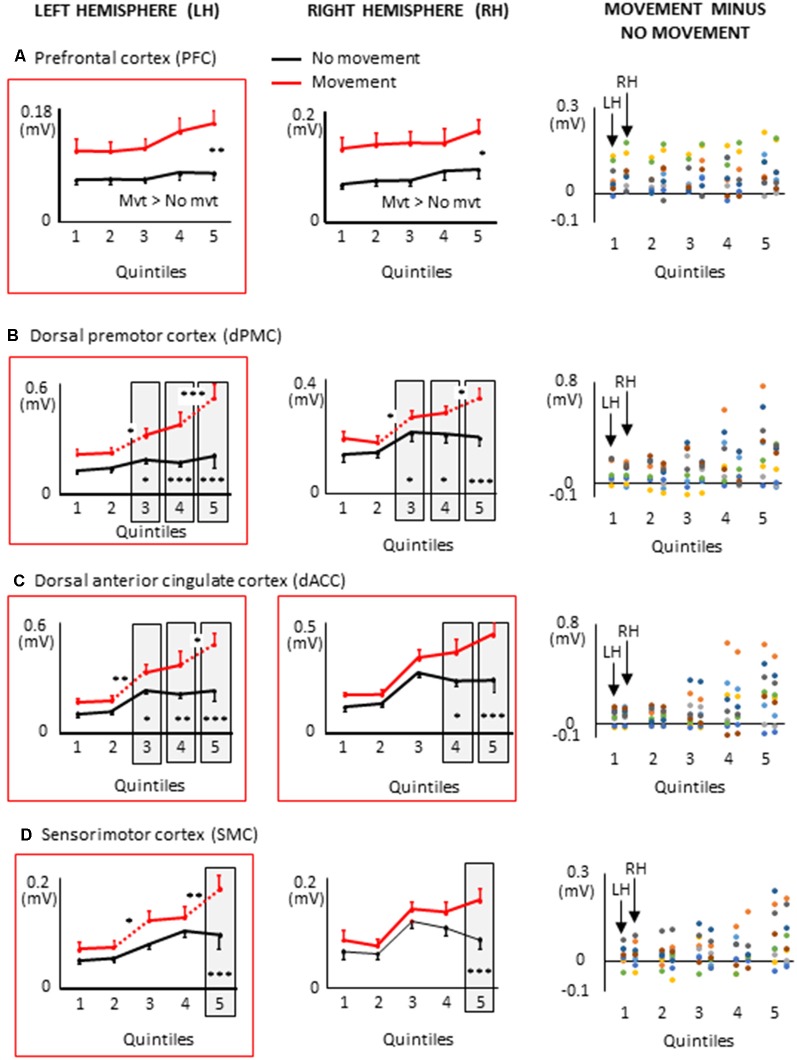
Mean activities recorded in the ROIs of the left and right frontal lobes (left and middle columns, respectively) showing a significant main effect of movement and a significant interaction between movement × quintile in Experiment 2 (delayed movements): i.e., prefrontal cortex, **(A)**; dorsal premotor cortex, **(B)**; Dorsal anterior cingulate cortex, **(C)**; sensorimotor cortex, **(D)**. The figure depicts only the results of the cortical regions that also showed significant effects in Experiment 1 (no delay) in either hemisphere (identified by red boxes). Error bars represent standard error of the mean. Dotted lines link consecutive quintiles showing significantly different activities (*p < 0.05, **p < 0.01, ***p < 0.001). Graphs of the right column depict the differences in activity between the Movement and No-movement conditions for all participants. Dots of the same color represent data from the same participant. Values greater than 0 (i.e., above X-axis) indicate that the participants showed greater activity in the Movement condition than in the No-movement conditions.

**Figure 8:**
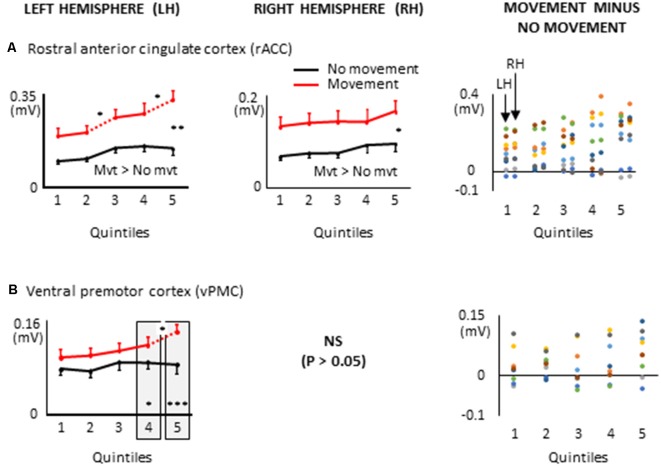
ROIs of the frontal lobe showing significant effects only when a 8 s delay was imposed between body motion and the arm movement: i.e., rostral anterior cingulate cortex, **(A)**; ventral premotor cortex, **(B)**. More specifically, the graphs show the mean activities recorded in the ROIs of the left and right hemispheres (left and middle columns, respectively) showing a significant main effect of movement and a significant interaction between movement × quintile in Experiment 2 (delayed movements). Error bars represent standard error of the mean. Dotted lines link consecutive quintiles showing significantly different activities (*p < 0.05, **p < 0.01, ***p < 0.001). Graphs of the right column depict the differences in activity between the Movement and No-movement conditions for all participants. Dots of the same color represent data from the same participant. Values greater than 0 (i.e., above the X-axis) indicate that the participants showed greater activity in the Movement condition than in the No-movement conditions. LH and RH indicate left and right hemispheres, respectively.

**Figure 9:**
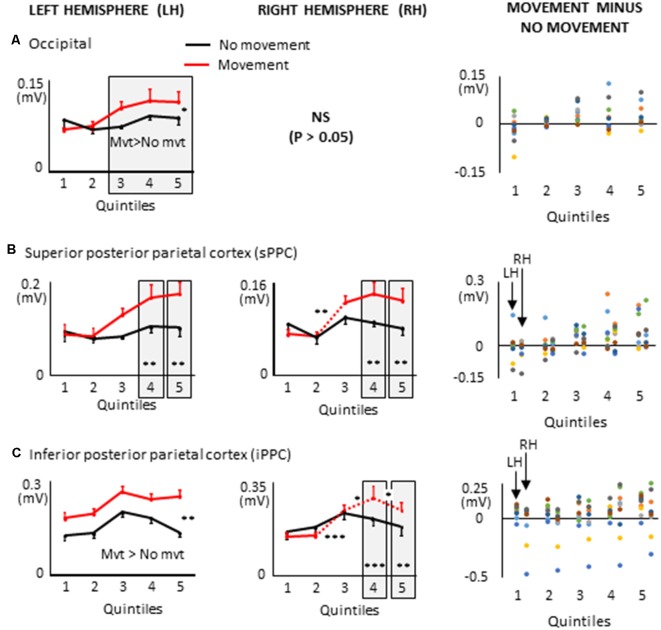
Mean activities recorded in the ROIs of the left and right hemispheres (left and middle columns, respectively) of the posterior cortex showing a significant main effect of movement and a significant interaction between movement × quintile in Experiment 2 (delayed movements): i.e., occipital cortex, **(A)**; superior posterior parietal cortex, **(B)**; inferior posterior parietal cortex, **(C)**. Error bars represent standard error of the mean. Dotted lines link consecutive quintiles showing significantly different activities (*p < 0.05, **p < 0.01, ***p < 0.001). Graphs of the right column depict the differences in activity between the Movement and No-movement conditions for all participants. Dots of the same color represent data from the same participant. Values greater than 0 (i.e., above the X-axis) indicate that the participants showed greater activity in the Movement condition than in the No-movement conditions. LH and RH indicate left and right hemispheres, respectively.

EEG processing was performed as in Experiment 1. The baseline used to compute the co-variance matrices in the delayed arm movement condition was set between −9.5 s to −10 s with respect to the auditory cue (i.e., before body rotation for all trials of all participants). In the control condition, the baseline was set −1.5 s to −2 s prior to the first of the two tones. Albeit spatial and sensorimotor processes most likely took place before the imperative signal, for each ROI, we compared the mean current amplitude computed in each quintile of movement reaction time with the mean current amplitude computed during similar five time-windows in the control condition. EMG reaction time and quintile durations are provided for all participants in Figure 6. On average, the EMG reaction times and quintile duration were 294 ± 59 ms and 59 ms, respectively. Statistical results of the 2 (Condition: delayed movement, control) × 5 (Quintile: 1–5) repeated measures ANOVAs and of the *post hoc* tests are presented in Figures 7–9 and Table 1, respectively.

## Results and Discussion

### Extended Task-Related Activities in the Frontal Lobe With Delayed Arm Movements

All frontal ROIs that showed greater activation during movement planning in Experiment 1 also showed additional activation during movement planning in the delayed movement condition (i.e., PFC, dPMC, SMC, dACC; see Figure 7). However, in contrast to Experiment 1, the increased activations of the PFC, dPMC and SMC in the arm movement condition were bilateral rather than being restricted to the hemisphere contralateral to the moving arm (i.e., left hemisphere). In the light of the temporal constraints imposed in the delayed movement condition, the sustained task-related activation of the right PFC might have been associated with both the maintenance of body rotation and spatial information in working memory (Courtney et al., 1998; Romo et al., 1999; Katsuki et al., 2014), and the shift from cue monitoring (i.e., self-motion cues) to cued arm response (Howe et al., 2013). The greater activation of the right dPMC observed throughout movement planning could have also contributed to the storage of spatial information during the imposed delay (Smith and Jonides, 1999). Supplementary activation was also found in the right SMC in the last quintile of the movement condition. This ipsilateral activity could reflect motor processes related to postural adjustments associated to the execution of the rapid upper limb movement (Massion, 1994; Kurtzer et al., 2005).

Other ROIs from the frontal lobe showed significant task-related activations during the delayed arm movement that were not observed in Experiment 1 (i.e., without an imposed delay; see Figure 8). This was the case of the rostral ACC (rACC) where current amplitude was found to be bilaterally greater throughout movement planning. This observation could be linked to visuo-spatial attention and exploratory processes that have been identified in the rACC (Corbetta et al., 1993; Kim et al., 1999; Amiez et al., 2012). Besides, the current amplitude of the rACC contralateral to the reaching hand progressively increased during movement planning, reaching its maximum value in the last quintile. This increased activation is consistent with studies demonstrating that the rACC plays an important role in the control of arm movements (Picard and Strick, 1996; Paus, 2001), notably those relying on working memory (Paus et al., 1998).

Task-related activations were also found in the left ventral premotor cortex (vPMC) when arm movements were delayed. More specifically, the vPMC showed greater activation in the movement condition than in the no-movement condition in the last two quintiles. The vPMC is an important area for space perception and activity in this area has been associated with the encoding of hand movements in extrinsic coordinates (Kakei et al., 2001; Rizzolatti et al., 2002). Thus, the increased vPMC activation observed towards the end of movement planning could reflect processes related to the transformation of the extrinsically encoded spatial goal of the movement into arm motor commands.

### Large Task-Related Activations in the Posterior Cortex During Delayed Arm Movements

Several ROIs from the posterior cortex showed larger activation during movement planning when arm movements were delayed after body rotations (see Figure 9). This was the case of the left occipital cortex which showed greater activation in the movement condition in the last three quintiles (i.e., last 60% of the movement planning). This observation is important when considering the frame of reference used for motor planning. Indeed, it suggests that despite the absence of visual information, a visual-type of spatial representation might have been used to plan the arm movement. This finding is consistent with studies showing that even in the absence of visual feedback, the occipital cortex can provide relevant information to the motor system (Singhal et al., 2013; Manson et al., 2019).

Both superior PPC showed greater current amplitudes in the last two quintiles of movement planning. Previous studies have found that in this area of the parietal lobe, movement planning is based on visual space representations (e.g., Andersen et al., 1997). Specifically, it has been shown that activity in superior PPC is associated with the computation of motor errors in extrinsic coordinates, even for movements produced in darkness (Darling et al., 2007; Medendorp et al., 2008; Filimon et al., 2009). Note that because the activation of the superior PPC did not significantly differ between the movement and no-movement conditions in the first three quintiles, the results obtained with the present protocol cannot provide support for the hypothesis that this region contributes to hold motor plans during delayed actions (e.g., Gnadt and Andersen, 1988).

The left and right inferior PPC showed greater activation throughout movement planning and in the last two quintiles, respectively. These activities could be associated to space encoding which is known to be performed relative to the arm and hand in the inferior PPC (Rozzi et al., 2008). The additional activation found in this area during movement planning could, therefore, be relevant for computing the motor vector in the superior PPC.

## General Discussion and Conclusion

The pattern of cortical activations found in the present study when the arm movements were triggered 8 s after the passive body rotations (Experiment 2) is consistent with the scheme that goal-directed arm movements produced after body motion was derived from a visually-based updated internal representation of the environment. These results are therefore coherent with the most widely adopted view that cognitive maps intervene in the organization of spatially-oriented behavior based on body motion information (Loomis et al., 1996; Klier and Angelaki, 2008; Mackrous and Simoneau, 2011, 2014; Medendorp, 2011). These cognitive representations would be particularly suited for storing relevant spatial information when the motor response is postponed after body motion (Bridgeman, 1991; Burgess, 2008). To our knowledge, these observations provide the first human electrophysiological evidence for the contribution of such cognitive processes for planning motor actions based on idiothetic information. These results, therefore, build on recent studies describing cortical activities evoked by body rotations (e.g., Gale et al., 2016) or activities strictly linked to spatial, non-motor updating processes (Gutteling and Medendorp, 2016; Gutteling et al., 2017; de Winkel et al., 2017).

Importantly, however, the spatio-temporal patterns of cortical activation revealed in the present study confer greater contribution of higher-order cognitive processes in movement planning when a delay is introduced between body motion and the arm motor response. Indeed, the results of Experiment 1 suggest that a more sensorimotor type of representation is responsible for organizing arm movements that are promptly triggered after body motion. Observations made in Experiment 1 are therefore consistent with studies suggesting that, despite being context-dependent (Keyser et al., 2017; Smith and Reynolds, 2017), the control of arm movements based on vestibular input can be largely independent of cognitive processes (Bresciani et al., 2005; Blouin et al., 2010; Guillaud et al., 2011; for review, see Blouin et al., 2015). Nonetheless, our observation that task-related activities were found in similar frontal areas in conditions with and without delay (e.g., dACC and dPM) suggests that the cognitive processes occurring in occipito-parietal regions did not supersede the frontal sensorimotor processes. Rather, our findings provide evidence that cognitive and sensorimotor processes contribute together for triggering delayed arm motor actions based on idiothetic information.

## Data Availability Statement

The datasets generated for this study are available on request to the corresponding author.

## Ethics Statement

The studies involving human participants were reviewed and approved by Laval University Biomedical Ethics Committee. The patients/participants provided their written informed consent to participate in this study.

## Author Contributions

JB, MS and LM designed the experiments, interpreted the data and wrote the manuscript. JB and MS performed the experiments. AS and GM analyzed the data. GM revised the manuscript. J-PP programmed the experiment.

## Conflict of Interest

The authors declare that the research was conducted in the absence of any commercial or financial relationships that could be construed as a potential conflict of interest.
